# Overuse of Short-Acting Beta-2 Agonists (SABAs) in Elite Athletes: Hypotheses to Explain It

**DOI:** 10.3390/sports10030036

**Published:** 2022-03-02

**Authors:** Nicolas Vertadier, Wojciech Trzepizur, Sébastien Faure

**Affiliations:** 1 Département Pharmacie, Faculté de Santé, University Angers, F-9000 Angers, France; vertadier.nico@hotmail.fr; 2 Mitovasc, University Angers, Inserm, CNRS, SFR ICAT, CHU Angers, F-49000 Angers, France; wotrzepizur@chu-angers.fr; 3 MINT, University Angers, Inserm, CNRS, SFR ICAT, F-49000 Angers, France

**Keywords:** short-acting beta-2 agonists, sport, asthma, exercise-induced bronchoconstriction, doping, overuse

## Abstract

The use of short-acting beta-2 agonists (SABAs) is more common in elite athletes than in the general population, especially in endurance sports. The World Anti-Doping Code places some restrictions on prescribing inhaled β2-agonists. These drugs are used in respiratory diseases (such as asthma) that might reduce athletes’ performances. Recently, studies based on the results of the Olympic Games revealed that athletes with confirmed asthma/airway hyperresponsiveness (AHR) or exercise-induced bronchoconstriction (EIB) outperformed their non-asthmatic rivals. This overuse of SABA by high-level athletes, therefore, raises some questions, and many explanatory hypotheses are proposed. Asthma and EIB have a high prevalence in elite athletes, especially within endurance sports. It appears that many years of intensive endurance training can provoke airway injury, EIB, and asthma in athletes without any past history of respiratory diseases. Some sports lead to a higher risk of asthma than others due to the hyperventilation required over long periods of time and/or the high environmental exposure while performing the sport (for example swimming and the associated chlorine exposure). Inhaled corticosteroids (ICS) have a low efficacy in the treatment of asthma and EIB in elite athletes, leading to a much greater use of SABAs. A significant proportion of these high-level athletes suffer from non-allergic asthma, involving the th1-th17 pathway.

## 1. Introduction

Regular physical activity is associated with better asthma control. Our society encourages people to take part in sports, which helps to reduce the incidences of chronic diseases and allows patients with these diseases to maintain a better quality of life. Five to ten minutes of exercise a day could reduce all-cause mortality by 30% [1,2,3,4,5].

Physical exercise has a beneficial effect on the quality of life of asthma patients [2,3,4]. However, there is a higher prevalence of the use of medication for the treatment of asthma, particularly short-acting beta-2 agonists (SABA), in elite athletes than in the general population.

Except for salbutamol, SABAs are prohibited by the World Anti-Doping Agency, without a therapeutic use exemption (TUE). Inhaled corticosteroids (ICS) are permitted [6].

Data collected among athletes at the Atlanta, Sydney, and Athens Olympic Games show significant use of short-acting adrenergic agonists in elite athletes (e.g., 15% of cyclists, 11% of swimmers, and 16% of speed skaters) [7].

This overconsumption involves only endurance sports as only 2.2% of tennis and handball and 1.1% of gymnastics athletes use them.

The objective of this review is to provide possible explanations for the overuse of SABA in the high-performance athlete.

## 2. The Shadow of Doping

The performance of athletes receiving treatment for asthma does not only seem to match, but rather exceed that of untreated athletes.

During the Sydney Summer and the Vancouver Winter Olympics, athletes being treated for asthma represented between 4% and 8% of the participating athletes; however, they made up between 5% and 16% of medal winners [8].

This high use of treatment appears to be due to a greater prevalence of asthma, particularly in endurance sports [9].

However, the fact that athletes seem to perform better when taking asthma treatments, and that they even outperform their colleagues without lung disease, raises questions regarding the reasons for this overuse.

### 2.1. Are SABAs Associated with a Doping Effect?

Many studies have investigated the possible doping effect of beta-adrenergic agonists.

The results of these studies vary depending on the protocol used. Time trials seem to show beneficial effects of inhaling beta-adrenergic agonists, whereas “time to exhausting” efforts show no effect. This could be due to a final-kick effect, or a possible beneficial effect on the sprinting abilities (maximum effort of 10–30 s) of salbutamol. The study by Hostrup et al. on the effects of acute and two-week administration of salbutamol on exercise performance, sprint of 30 s repeatability, and muscle strength in athletes without asthma and exercise-induced bronchoconstriction (EIB) showed only an improvement in sprinting ability. Two studies by Merlini et al. gave different results. One showed no effect after inhalation of 1600 micrograms of salbutamol on sprinting performance in footballers (30 m). However, in another study, there was an increase in sprinting ability over 30 m with LABA inhalation (200 micrograms of salmeterol or 24 micrograms of formoterol) compared to a placebo. In the large majority of studies, there was no improvement in the performance of a healthy athlete with beta-adrenergic agonist inhalation. An improvement in lung function in athletes with or without asthma, particularly in forced expiratory volume (FEV1), is found with inhaled SABA or long-acting beta-2 agonists (LABA). It cannot be concluded that there is a significant doping effect of beta-2 adrenergic agonists [7,10,11,12,13,14,15].

### 2.2. So, Why This Overconsumption?

Is it because champion athletes train harder than other athletes, following the adage that “the harder I train, the better I perform”, and are therefore more likely to report asthma?

## 3. Epidemiology

Several epidemiological studies have shown an abnormally high and increasing prevalence of asthma in professional athletes compared to the general population [16,17].

Asthma prevalence was at 6.7% in the general French population in 2006 whereas it varied from 15 to 20% in high-level athletes practicing endurance sports [18].

Similarly, the prevalence of asthma among American and German Olympic athletes increased from 11.2% in 1984 to 15.3% in 1996 and to 17% in 2007. During that time, the prevalence was estimated to be between 4 and 7% in the American and German populations [19,20].

Exercise-induced bronchoconstriction mainly affects people with asthma, but it also affects those without chronic asthma, including elite athletes. The term “exercise-induced” bronchoconstriction is used rather than “asthma-induced” because the former term does not imply underlying asthma. The symptoms of EIB are the same as those of chronic asthma, and they can vary greatly and are only triggered by exercise. EIB can be asymptomatic and only cause a decrease in lung function. EIB in athletes is very often asymptomatic or of mild intensity [7,21,22].

EIB is reported to occur in 90% of people with chronic asthma, especially in those with severe or poorly controlled forms [10,23]. In contrast, between 5 and 20% of the general population are estimated to experience EIB. The prevalence in athletes is higher than in the general population and is estimated to be between 30 and 70% [22,24,25].

Age and gender are not predisposing factors for asthma or EIB in elite athletes. The type of effort made is a better predictor of EIB. Indeed, asthma or EIB in elite athletes will preferentially affect those practicing endurance sports, especially in hot or cold climates [6].

Consequently, the use of SABAs by athletes in the winter and summer Olympic Games from 2002 to 2010 was higher in endurance sports such as cycling (17.2%) or Nordic combined (12.9%) than in ski jumping (3.1%) or boxing (1.7%) [9]. Harmful substances found in inhaled air, added to heavy ventilation, might increase the probability of asthma. For example, chloramine is known to be harmful to the airways, which might explain why synchronized swimming is affected by asthma [8]. Similarly, skaters have a high prevalence of respiratory pathologies that might be explained by the quality of the air inhaled in skating rinks due to ice resurfacing machines, which produce carbon monoxide, nitrogen dioxide, and fine and ultra-fine particles [26,27].

The prevalence of asthma is higher in elite athletes than in the general population, particularly in endurance sports. This is also the case for EIB. Chronic asthma in the elite athlete and especially EIB, which can be asymptomatic, is often mild in intensity [28]. Prior to 2019, Global Initiative for Asthma (GINA) recommended the administration of SABAs alone for level-1 asthma and stepping up the therapy if not controlled.

## 4. A Low Use of Inhaled Corticosteroids and Therefore a Greater Use of SABA

Of the athletes taking SABAs, the number who are also taking ICS has steadily increased from the 2004 Athens Olympics (69.9%) to the 2008 Beijing Olympics (87.2%). However, at the 2010 Vancouver Olympics, this percentage decreased sharply (75.3%) [9].

Daily use of ICS remains the most effective treatment for allergic asthma. They are the most effective anti-inflammatory agents of the airways and give better control of underlying asthma. Their use results in fewer emergency treatments composed of a SABA alone or a LABA-ICS [29].

There is a synergistic effect between ICS and LABA [29]. The use of a beta-2 adrenergic agonist alone is not recommended as there is an increased risk of poor asthma control and exacerbations [30,31].

In allergic asthma, ICS act against bronchial remodeling and therefore against bronchial hyperreactivity [32]. In athletes with isolated EIB or mixed asthma, inhaled glucocorticoids should also act against bronchial remodeling.

### 4.1. So Why the Heavy Use of Beta Agonists Instead of ICS?

#### 4.1.1. Variable Effectiveness

Oral corticosteroids provide significant improvement in FEV1 (which corresponds to the volume of air exhaled during the first second of a so-called “forced” exhalation, following a deep inhalation) for some chronic asthmatic patients [23,33]. The severity of asthma alone cannot explain the resistance to, or the effectiveness of, corticoids.

In skiers, ICS have a similar anti-inflammatory or symptom-reduction effect to placebo, but they induce a significant improvement in FEV1 [34].

In other chronic asthmatics during the birch pollen season, the use of ICS contributed to a significant reduction in the number of inflammatory cells [32].

The different responses of the inflammatory process to inhaled corticosteroids in “skiing asthma” and “allergic asthma” may suggest that the stimulation of the inflammatory response is different in these two asthma phenotypes.

Similarly, the efficacy of ICS on frequency and intensity of EIB (isolated or associated with asthma) will also differ between patients.

Chronic ICS treatments have long and repeatedly demonstrated their effectiveness in reducing the frequency and intensity of EIB in the general population [35].

However, few studies have been conducted in elite athletes on the action of ICS on EIB.

The studies performed to date have not been able to show any clear beneficial effect of ICS on airway inflammation. The improvement in FEV1 is mostly attributed to the cessation of sports activity [34].

A study by Tsukioka K. et al. also identified a category of subjects who did not respond well to ICS treatment alone but responded to treatment with the ICS/LABA combination. Athletes with allergic asthma were the group that responded best to different treatments [36].

#### 4.1.2. Immunologic Profile and Inflammatory Responses in Asthma

Asthma was originally thought to have a single inflammatory mechanism, both in athletes and in non-athletes. The airway immune process leading to asthma symptoms was thought to be solely due to the th2 pathway. Asthma caused by the th2 pathway is allergic asthma. Analyses of bronchial biopsies and bronchoalveolar lavage fluids taken from most asthmatic patients showed elevated levels of L T CD 4 and markers that indicate an immune mechanism of this th2 pathway. They also showed high levels of eosinophils, mast cells, IL4, IL 5, IL 9, and IL 13. This causes an increased production of EGI by lymphocytes, thus releasing mediators responsible for asthma symptoms and the development of chronicity including: histamine, prostaglandins (notably PGD2), leukotriene (notably LT C4, D4, and E4), and major basic proteins [37,38,39,40,41,42].

However, thanks to several studies conducted over the last few years, it is now known that this is not the only inflammatory mechanism. We can speak of asthma phenotype, which depends on the higher or lower expression of each immunological pathway in the patient’s asthmatic pathology, and on their response to treatments [43,44].

Nevertheless, in all asthma phenotypes, whether mild to moderate, there is a certain level of collagen and tenascin deposited on the basement membrane, as well as hypertrophy and hyperplasia of the smooth muscles of the airways and airway goblet cell hyperplasia [31].

#### 4.1.3. Asthma th1 and th17

Several studies in patients with severe asthma, and/or a poor response to corticosteroids, show elevated levels of cytokines and cells not representative of the th2 pathway but rather of the th1 and th17 pathways. These include gamma interferon, IL8, and IL-17 with significant neutrophil expression, which is known to play a role in airway remodeling leading to bronchial hyper-responsiveness (BHR) and chronic asthma. In these patients, the pathology is very often late-onset, occurring after childhood [38,39,40,41].

Aggression of the airways induces an inflammatory response of the organism mediated by the th1-th17 pathway. The CD4 cells of the th1 pathway secrete the interleukin interferon (IFN) gamma.

The IFN gamma is able to stimulate phagocytic activity by activating macrophages. The CD4 cells of the th17 pathway induce mainly a secretion of interleukins IL-17, IL-21, and IL-22. The IL-17 stimulates the production of pro-inflammatory cytokines such as IL-6, IL-1, and TNF alpha.

The IL-17 and IL-22 are associated with the stimulation of the production of granulopoietic factors such as IL-8 and G-CSF, notably by bronchial epithelial cells. These two factors favor proliferation and induce the recruitment of neutrophils. The Il-21 also induces the production of IL-8 while IL-6 and IL-17 increase mucus production. Neutrophils release chemotactic factors to attract monocytes and macrophages. They have an important role in the defense of the organism with their antimicrobial activity through the generation of reactive oxygen species (ROS), the activation of protease, and the establishment of neutrophil extracellular traps (NET). In addition to the production of TGF-beta, metalloprotease (MMP9), and elastase, neutrophils have a role in airway remodeling by degrading the extracellular matrix and the epithelial barrier. The elastase produced by neutrophils contributes to inhibiting anti-protease (TIMP). The IFN gamma inhibits anti-elastase (SLP1), which implies the persistence of proteases such as metalloprotease produced by neutrophils. This creates an imbalance between the proteases that degrades the extracellular matrix and the anti-proteases that inhibit proteases. This is a possible explanation for why we see a thickening of the basement membrane with increased deposition of extracellular matrix proteins like collagen and tenascin. This leads to the development of airway remodeling and to chronic asthma [38,39,40,41].

At the systemic level, CS will cause suppression of the th1-th17 immune axis and a shift towards th2-mediated immunity. This effect does not apply to all specific compartments of the human body. At the pulmonary level, the opposite is true. There is a suppression of the th2 pathway and upregulation of the th1-th17 voice [23,33,45].

There are explanations for the lack of efficacy of CS in th1-th17 asthma, but several hypotheses remain to be explored [23,30,31,32,33,34,35,36,37,38,39,40,41,42,43,44,45,46].

#### 4.1.4. To Which Phenotype Does an Athlete’s Asthma and EIB Asthma Belong?

The onset of asthma in athletes occurs later than in the general population. This pathology appears during or after adolescence and does not correspond to the usual allergic asthma that develops mainly in childhood. Years of intensive endurance training could be a trigger for exercise-induced asthma.

A study of athletes at the 2006 Turin Olympic Games showed that only 32.1% of athletes using a beta-2 adrenergic agonist had the onset of their asthma during childhood. In 48.7% of these athletes, asthma occurred after the age of 20 [47]. Similarly, in skiers, asthma often appears in adolescence or later, but it is rare in childhood [48,49].

Numerous studies show that the chronic and late-onset asthma that many athletes suffer from is mixed, involving the th2 and th1-th17 pathways.

According to the article by Carlsen K-H et al. published in 2012, these athletes have a high number of neutrophil cells in their sputum, and the higher the training load, the higher the number of neutrophil cells. Higher levels of interleukins 8, 17, and 22 are also found in their sputum as is CC16. It is interesting to note that neutrophilic inflammation does not respond to ICS [50].

An increase in neutrophil and lymphocyte levels is found in professional swimmers, whether or not they have asthma [51].

Chlorine derivatives will injure the airways and trigger neutrophil inflammation, an expression of the th1-th17 pathways. When these swimmers are asthmatic, the inflammation is mixed and involves a th1-th17 pathway but also a th2 pathway immune mechanism [52].

In skiers, whether asthmatic or not, high levels of lymphocytes, eosinophil macrophages, and neutrophils are observed [53,54].

The tenascin composition of the basement membrane is significantly higher in asthmatic individuals and skiers than in healthy non-skiers, Ref. [55] reflecting a process of aggression and repair of the airway epithelium, and thus airway remodeling.

Asthma in high-level skiers involves an inflammatory mechanism of the th1-th17 and th2 pathways [34].

These same high levels of neutrophils and eosinophils are also found in ice hockey players, showing a mixed expression of the th2 and th1-th17 pathways [27].

#### 4.1.5. Pathophysiology of Sports Asthma

The late-onset of asthma in elite athletes is explained by the fact that over the years, high-intensity training causes recurrent injuries to the bronchial epithelium, leading to bronchial hyper-responsiveness (BHR). This is an increased sensitivity of the airways to physical or chemical stimuli that will cause excessive narrowing of the lumen in these airways [56,57,58].

BHR is present in subjects with or without chronic asthma. In individuals without associated chronic asthma, BHR will trigger EIB.

The presence of BHR in an individual might lead to the development of chronic asthma, whether allergic or not.

Sustained exercise requiring increased oral respiration will lead to dehydration on the surface of the airway epithelium. Hyperventilation in dry, cold air will exacerbate this phenomenon and therefore result in a higher frequency of BHR. Dehydration will make the bronchial epithelium prone to injury.

These injuries, which lead to the th17 inflammation, will trigger microvascular leakage of bulk plasma from the surface of the airways to provide the injured epithelium with substances to regenerate itself and restore fluid at the surface.

Plasma contains substances that will alter the growth and contractile properties of smooth muscle cells.

In endurance athletes, this process of injury and repair will be repeated several times over numerous sessions. After several exposures to molecules contained in the plasma, a change in the contractile properties of the smooth muscles appears, and they become hypersensitive, leading to bronchial hyper-responsiveness (BHR).

Because these smooth muscles of the airways are hypersensitive, the release of inflammatory mediators caused by the inhalation of allergens or substances harmful to the bronchial tree will trigger bronchoconstriction. From then on, isolated EIB will set in, and it may be accompanied by chronic asthma at a later stage.

The current leading theory for explaining EIB is the osmolar theory.

On the surface of the epithelial cells, there is hyperosmolarity of the surface fluid, which will extend to the epithelial cells and the submucosa of the airways.

The hyperosmolar stimulus caused by the loss of water on the surface of the airways will trigger the release of IL8, which leads to the proliferation of neutrophils, as well as prostaglandins (PGD2), leukotrienes (LTE4), and histamine. This will cause bronchoconstriction and increase vascular permeability. Hyperosmolarity will also stimulate neuronal cells to release tachykinins. Tachykinins are more effective if the epithelium is damaged because they are less degraded.

The destruction of epithelial cells will decrease the levels of prostaglandin PGE2, which protects against asthma.

More sensitive, smooth muscle cells will produce more chemokines to attract mast cells.

The mast cells will infiltrate the smooth muscle of the airways; they are more likely to be present in the tissues of allergen-sensitized individuals and in those with chronic asthma [37,50,59,60].

An altered epithelial barrier will expose sensory nerve endings to exogenous particles and endogenous inflammatory mediators.

The goblet cells will replace the destroyed epithelial cells, leading to increased sputum levels [58,61].

Several studies have shown that elite athletes have a greater risk of developing allergies—they have higher levels of allergic rhinitis, allergic rhinoconjunctivitis, and dermatitis.

The prevalence of allergic rhinitis and food allergies among Italian athletes during the Sydney and London Olympic Games was 26.2% and 7.1%, respectively [62,63]. By comparison, the prevalence of allergic rhinitis is estimated to be 15% in the European population according to INSERM and that of food allergy is 1–3% in the world population according to the WHO [64,65]. In atopic subjects, playing a sport requiring hyperventilation, years of high-intensity training resulting in the development of BHR, and repeated exposure to allergens which could promote the development of allergic rhinitis and/or allergic asthma [59,63]. The non-asthmatic atopic athlete may develop an inflammatory response (and therefore asthma) more easily, as he or she is likely to have more inflammatory mediators, IGEs, and mast cells than the non-atopic, non-asthmatic athlete.

The various attacks on the epithelium of the bronchial tracts, the oxidative lesions caused by hyperventilation, combined with inhaled air that has a high concentration of chlorine derivatives (or which is polluted, cold, and dry) will trigger a defense mechanism in the body. This defense mechanism involves a th2 type immune response, and especially a th1-th17 type immune response, represented by the increased levels of neutrophils. These repeated aggressions lead to BHR and mixed chronic asthma of th2 and th1-th17 pathways in high-level men and women athletes.

**Figure 1:**
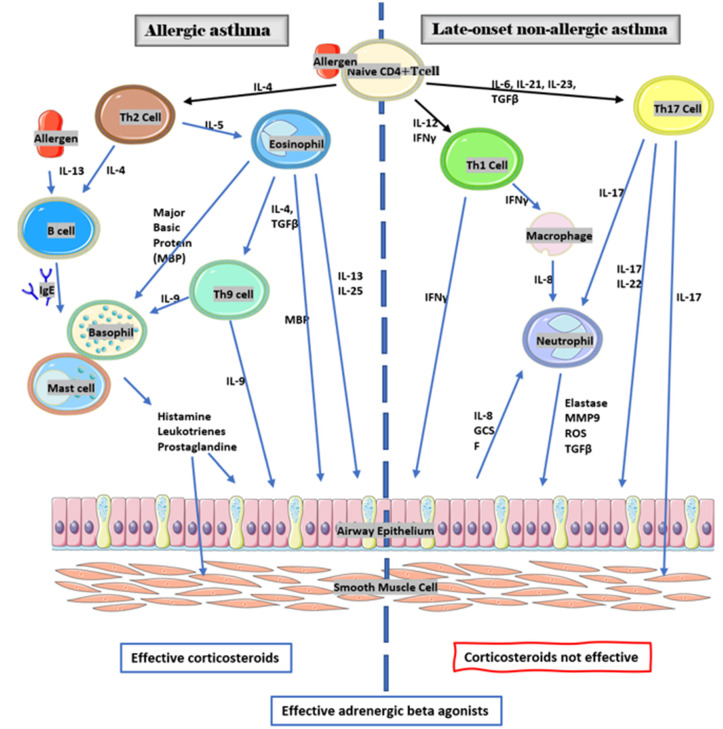
Physiopathology of asthma [37,38,39,40,41,42,66].

Two asthma phenotypes are reported in elite athletes (Figure 1).

“Classical” asthma begins in childhood or later and involves BHR of allergic origin in athletes who are usually atopic.

“Late-onset” asthma begins after adolescence, during the athlete’s career, and is due to hypersensitization of the airways caused by repeated high-intensity training.

The black arrows indicate that when the antigen is presented to naïve CD4 T helper cells, it triggers their differentiation into th2, th1, or th17 cells. The immunological pathway depends on the type of antigen and the composition of the microenvironment of the naïve CD4 T helper cell. The blue arrows indicate that one cell type acts via molecules on another cell type.

#### 4.1.6. Are Adrenergic Agonist Betas Effective in Mixed Asthma th2 th1–17?

Several studies have demonstrated the efficacy of SABAs and LABAs for treating asthma in elite athletes. In high-performance skiers with asthma, inhalation of a SABA or LABA significantly improved FEV1 in the minutes following inhalation, during and after exercise [67].

Beta-adrenergic agonists could be effective at treating and preventing EIB in the high-performance athlete [7,29]. Their efficacy may be due to their bronchodilator effects but also their effects on acute inflammation, even if overuse of SABAs is known to be associated with increased risk of exacerbation and mortality in asthma [68].

SABAs and LABAs inhibit the release of inflammatory mediators (histamine or interleukins) by mast cells, LTCD4 th2, basophils, monocytes, etc. This effect must be qualified because although it is well established in vitro, it is much less so in vivo [69,70]. They decrease micro vascular permeability and reduce the release of bulk plasma that causes bronchial hyper-reactivity by inhibiting the formation of endothelial spaces [37].

The effect of beta-agonists on chronic inflammation is not yet established [70].

However, adrenergic beta-agonists also affect neutrophil activation. They do this by inhibiting the adhesion of neutrophils to epithelial cells and inhibiting their activation. Chronic treatment of mild asthma with a LABA reduces the number of neutrophils in the bronchi. Adrenergic agonist beta-agonists are also believed to accelerate neutrophil apoptosis.

The study by Perttunen, H. et al. (2008) showed a significant reduction in the neutrophil count with salbutamol or formoterol, but no effect with salmeterol. This study also showed that the opposite is true when combining an adrenergic beta-agonist and an inhaled corticosteroid (Budesonide and Fluticasone): in that case, there is no apoptotic effect on neutrophils [71].

The study by Anderson R et al. showed that the LABAs formoterol and indacaterol are more effective in reducing neutrophil activity than a SABA, which in this study was salbutamol [72].

The lack of efficacy of ICS in mixed phenotype asthma may explain why it is more difficult to control in athletes, and thus why they turn to SABAs (which remains effective) more often than non-athletes.

## 5. Very High Frequency of Use in Athletes

Unfortunately, a tolerance effect may occur [37,73,74].

Desensitization of beta-agonist receptors will affect mast cells and lymphocytes. In the case of eosinophilic lymphocytes, the process is slower for smooth muscle cells of the respiratory tract [70].

The anti-inflammatory effect of beta-2 agonists is lost faster than its bronchodilator effect. This phenomenon occurs due to chronic overuse (i.e., several times a day over two weeks for albuterol or salbutamol, or daily use of salmeterol over one month) [37,73,74].

Because of this tolerance, the patient will need even to administer higher doses and/or increase the frequency of administration.

More infrequent use reduces this phenomenon. The efficacy of formoterol inhalation remains unchanged over time if it is used three times or less per week [29].

Concomitant use of corticosteroids does not affect this loss of efficacy, whether for a SABA or LABA [29,75,76].

## 6. Summary

The doping theory cannot be used to explain the overconsumption of SABAs in elite athletes. Several other reasons for their high use include the fact that there is a very high prevalence of EIB and asthma in this population. These pathologies are generally mild in intensity, and prior to 2019, GINA recommended the use of a SABA alone for patients with tier-1 asthma.

ICS provide better asthma control and results in less frequent use of SABAs. However, ICS have been shown to have limited efficacy in the th2 and th1-th17 mixed asthma phenotype, which is late-onset and the most common asthma phenotype found in elite athletes. This lack of efficacy may explain their greater use of beta-adrenergic agonists, which remain effective regardless of an asthma phenotype. The high frequency of administration may be explained by the tolerance effect.

Nevertheless, ICS remain useful in the prevention and limitation of the aggravation of mixed asthma by decreasing bronchial remodeling. GINA recommends them in mild asthma (very present in athletes) or in EIB, which can generally be reduced with a maintenance ICS [28].

Therefore GINA recommendations are the combination of ICS with LABA or SABA, whether it is allergic or mixed asthma [28].

## 7. Perspective

Where is the line between pathology and the physiological limits of the athlete?

The prescription of asthma medication in athletes is based on clinical symptoms and HBR/EIB test positivity. EIB is under-diagnosed; if all the athletes were tested, 30 to 70% of athletes may test positive [22,25,77].

This lack of diagnosis of EIB is because the condition may be asymptomatic, or its symptoms may be so mild that the athlete will consider it normal or due to a drop in fitness. High-intensity training over many years will lead to the development of these conditions. Many athletes will fortunately not develop asthma or EIB. The body’s ability to manage this oxidative stress is important and depends on the individual, as well as the means used to prevent the development of these diseases.

Asthma or isolated episodes of EIB may be triggered by factors other than intense training. In megacities such as Beijing, the very high concentrations of pollutants will act directly on the neuroreceptors of the airways and have an irritant and inflammatory effect, provoking these symptoms [78].

Every individual will reach the limits of his body’s defense capacities when they reach a certain frequency of training, or if they train under certain conditions (which depend on the number of harmful particles in the air). This will trigger a respiratory pathology.

## Data Availability

Not applicable.

## References

[1] Alentorn-Geli E., Samuelsson K., Musahl V., Green C., Bhandari M., Karlsson J. (2017). The Association of Recreational and Competitive Running With Hip and Knee Osteoarthritis: A Systematic Review and Meta-analysis. J. Orthop. Sports Phys. Ther..

[2] Lang J.E. (2019). The impact of exercise on asthma. Curr. Opin. Allergy Clin. Immunol..

[3] Wen C.P., Wai J.P.M., Tsai M.K., Yang Y.C., Cheng T.W.D., Lee M.C., Chan H.T., Tsao C.K., Tsai S.P., Wu X. (2014). Minimal Amount of Exercise to Prolong Life. JACC Cardiol..

[4] Eichenberger P.A., Diener S.N., Kofmehl R., Spengler C. (2013). Effects of Exercise Training on Airway Hyperreactivity in Asthma: A Systematic Review and Meta-Analysis. Sports Med..

[5] Coëtmeur D., Parrat E., Nocent-Ejnaini C., Magiapan G., Prud’homme A., Oster J.-P., Appere de Vecchi C., Maurer C., Raherison C., Debieuvre D. (2020). Activité physique et asthme sévère: Résultats de l’étude FASE-CPHG. Rev. Des Mal. Respir..

[6] (2021). Béta-2-agonistes|World Anti-Doping Agency. https://www.wada-ama.org/fr/content/liste-des-interdictions/interdites-en-permanence/bta-2-agonistes.

[7] Carlsen K.H., Anderson S.D., Bjermer L., Bonini S., Brusasco V., Canonica W., Cummiskey J., Delgado L., Del Giacco S.R., Drobnic F. (2008). Exercise-induced asthma, respiratory and allergic disorders in elite athletes: Epidemiology, mechanisms and diagnosis: Part I of the report from the Joint Task Force of the European Respiratory Society (ERS) and the European Academy of Allergy and Clinical Immunology (EAACI) in cooperation with GA2LEN. Allergy.

[8] Fitch K.D. (2016). The World Anti-Doping Code: Can you have asthma and still be an elite athlete?. Breathe.

[9] Fitch K.D. (2012). An overview of asthma and airway hyper-responsiveness in Olympic athletes. Br. J. Sports Med..

[10] Koch S., Karacabeyli D., Galts C., Maclnnis M.J., Sporer B., Koehle M. (2015). Effects of inhaled bronchodilators on lung function and cycling performance in female athletes with and without exercise-induced bronchoconstriction. J. Sci. Med. Sport.

[11] Merlini M., Whyte G., Marcora S., Loosemore M., Chester N., Dickinson J. (2019). Improved Sprint Performance With Inhaled Long-Acting Β2-Agonists Combined with Resistance Exercise. Int. J. Sports Physiol. Perform..

[12] Kalsen A., Hostrup M., Backer V., Bangsbo J. (2016). Effect of formoterol, a long-acting β2-adrenergic agonist, on muscle strength and power output, metabolism, and fatigue during maximal sprinting in men. Am. J. Physiol. Regul. Integr. Comp. Physiol..

[13] Van Baak M.A., de Hon O.M., Hartgens F., Kuipers H. (2004). Inhaled Salbutamol and Endurance Cycling Performance in Non-Asthmatic Athletes. Int. J. Sports Med..

[14] Merlini M., Beato M., Marcora S., Dickinson J. (2019). The Effect of 1600 μg Inhaled Salbutamol Administration on 30 m Sprint Performance Pre and Post a Yo-Yo Intermittent Running Test in Football Players. J. Sports Sci. Med..

[15] Hostrup M., Kalsen A., Auchenberg M., Bangsbo J., Backer V. (2014). Effects of acute and 2-week administration of oral salbutamol on exercise performance and muscle strength in athletes. Scand. J. Med. Sci. Sports.

[16] Helenius I.J., Tikkanen H.O., Haahtela T. (1997). Association between type of training and risk of asthma in elite athletes. Thorax.

[17] Helenius I.J., Rytilä P., Metso T., Haahtela T., Venge P., Tikkanen H.O. (1998). Respiratory symptoms, bronchial responsiveness, and cellular characteristics of induced sputum in elite swimmers. Allergy.

[18] Afrite A., Allonier C., Com-Ruelle L., Le Guen N. (2011). IRDES. L’asthme en France en 2006: Prévalence, Contrôle et Déterminants.

[19] Voy R.O. (1986). The U.S. Olympic Committee experience with exercise-induced bronchospasm, 1984. Med. Sci. Sports Exerc..

[20] Thomas S., Wolfarth B., Wittmer C., Nowak D., Radon K., GA2LEN-Olympic study-Team (2010). Self-reported asthma and allergies in top athletes compared to the general population—Results of the German part of the GA2LEN-Olympic study 2008. Allergy Asthma Clin. Immunol..

[21] Poussel M., Chenuel B. (2010). Bronchoconstriction induite par l’exercice sans asthme associé chez l’athlète: Physiopathologie, diagnostic et prise en charge spécifique. Rev. Malad. Respir. Actual.

[22] Aggarwal B., Mulgirigama A., Berend N. (2018). Exercise-induced bronchoconstriction: Prevalence, pathophysiology, patient impact, diagnosis and management. NPJ Prim. Care Respir. Med..

[23] Leung D.Y.M., Martin R.J., Szefler S.J., Sher E.R., Ying S., Kay A.B., Hamid Q. (1995). Dysregulation of interleukin 4, interleukin 5, and interferon 1 gene expression in steroid-resistant asthma. J. Exp. Med..

[24] Weiler J.M., Anderson S.D., Randolph C., Bonini S., Craig T., Pearlman D., Rundell K., Silvers W., Storms W., Bernstein D.I. (2010). Pathogenesis, prevalence, diagnosis, and management of exerciseinduced bronchoconstriction: A practice parameter. Ann. Allergy Asthma Immunol..

[25] Parsons J.P., Craig T.J., Stoloff S.W., Hayden M.L., Ostrom N.K., Eid N.S., Colice G.L. (2011). Impact of exercise-related respiratory symptoms in adults with asthma: Exercise-Induced Bronchospasm Landmark National Survey. Allergy Asthma Proc..

[26] Rundell K.W. (2003). High Levels of Airborne Ultrafine and Fine Particulate Matter in Indoor Ice Arenas. Inhal. Toxicol..

[27] Lumme A., Haahtela T., Öunap J., Rytilä P., Obase Y., Helenius M., Remes V., Helenius I. (2003). Airway inflammation, bronchial hyperresponsiveness and asthma in elite ice hockey players. Eur. Respir. J..

[28] (2021). Global Strategy for Asthma Management and Prevention. www.ginathma.org.

[29] Parsons J.P., Hallstrand T.S., Mastronarde J.G., Kaminsky D.A., Kaminsky D.A., Rundell K.W., Hull J.H., Storms W.W., Weiler J.M., Cheek F.M. (2013). An Official American Thoracic Society Clinical Practice Guideline: Exercise-induced Bronchoconstriction. Am. J. Respir. Crit. Care Med..

[30] Leung D.Y., Bloom J.W. (2003). Update on glucocorticoid action and resistance. J. Allergy Clin. Immunol..

[31] Newton R., Giembycz M.A. (2016). Understanding how long-acting β2-adrenoceptor agonists enhance the clinical efficacy of inhaled corticosteroids in asthma—An update. Br. J. Pharmacol..

[32] Laitinen A., Altraja A., Kampe M., Linder M., Virtanen I., Laitinen L.A. (1997). Tenascin is increased in airway basement membrane of asthmatics and decreased by an inhaled steroid. Am. J. Respir. Crit. Care Med..

[33] Naseer T., Minshall E.M., Leung D.Y., Laberge S., Ernst P., Martin R.J., Hamid Q. (1997). Expression of IL-12 and IL-13 mRNA in asthma and their modulation in response to steroid therapy. Am. J. Respir. Crit. Care Med..

[34] Sue-Chu M., Karjalainen E.M., Laitinen A., Larsson L., Laitinen L.A., Bjermer L. (2000). Placebo-controlled study of inhaled budesonide on indices of airway infammation in bronchoalveolar lavage fuid and bronchial biopsies in cross-country skiers. Respir. Int. Rev. Thorac. Dis..

[35] Trigg C.J., Manolitsas N.D., Wang J., Calderon M.A., McAulay A., Jordan S.E., Herdman S.E., Jhalli N., Duddle J.M., Hamilton S.A. (1994). Placebo controlled immunopathologic study of four months of inhaled corticosteroids in asthma. Am. J. Respir. Crit. Care Med..

[36] Tsukioka K., Koya T., Ueno H., Hayashi M., Sakagami T., Hasegawa T., Arakawa M., Suzuki E., Kikuchi T. (2017). Phenotypic analysis of asthma in Japanese athletes. Allergol. Int..

[37] Anderson S.D., Kippelen P. (2008). Airway injury as a mechanism for exercise-induced bronchoconstriction in elite athletes. J. Allergy Clin. Immunol..

[38] Durrant D.M., Metzger D.W. (2010). Emerging Roles of T Helper Subsets in the Pathogenesis of Asthma. Immunol. Investig..

[39] Luckheeram R.V., Zhou R., Verma A.D., Xia B. (2012). CD4+T Cells: Differentiation and Functions. Clin. Dev. Immunol..

[40] Busse W.W., Lemanske R.F. (2001). Asthma. N. Engl. J. Med..

[41] Ray A., Kolls J. (2017). Neutrophilic Inflammation in Asthma and Association with Disease Severity. Trends Immunol..

[42] Akuthota P., Wang H.B., Spencer L.A., Weller P.F. (2008). Immunoregulatory roles of eosinophils: A new look at a familiar cell. Clin. Exp. Allergy.

[43] Wenzel S.E. (2012). Asthma phenotypes: The evolution from clinical to molecular approaches. Nat. Med..

[44] Moore W.C., Meyers D.A., Wenzel S.E., Teague W.G., Li H., Li X., D’Agostino R., Castro M., Curran-Everett D., Fitzpatrick A.M. (2010). Identification of Asthma Phenotypes Using Cluster Analysis in the Severe Asthma Research Program. Am. J. Respir. Crit. Care Med..

[45] Elenkov I.J. (2004). Glucocorticoids and the Th1/Th2 Balance. Ann. N. Y. Acad. Sci..

[46] Corrigan C.J., Brown P.H., Barnes N.C., Szefler S.J., Tsai J.J., Frew A.J., Kay A.B. (1991). Glucocorticoid Resistance in Chronic Asthma: Peripheral Blood T Lymphocyte Activation and Comparison of the T Lymphocyte Inhibitory Effects of Glucocorticoids and Cyclosporin A. Am. Rev. Respir. Dis..

[47] Fitch K.D. (2006). β2-Agonists at the Olympic Games. Clin. Rev. Allergy Immunol..

[48] Norqvist J., Eriksson L., Söderström L., Lindberg A., Stenfors N. (2015). Self-reported physician-diagnosed asthma among Swedish adolescent, adult and former elite endurance athletes. J. Asthma.

[49] Eklund L.M., Irewall T., Lindberg A., Stenfors N. (2018). Prevalence, age at onset, and risk factors of self-reported asthma among Swedish adolescent elite cross-country skiers. Scand. J. Med. Sci. Sports.

[50] Carlsen K.H. (2012). Sports in extreme conditions: The impact of exercise in cold temperatures on asthma and bronchial hyper-responsiveness in athletes. Br. J. Sports Med..

[51] Moreira A., Delgado L., Palmares C., Lopes C., Jacinto T., Rytilä P., Silva J.A., Gastel-Branco M.G., Haahtela T. (2008). Competitive swimmers with allergic asthma show a mixed type of airway inflammation. Eur. Respir. J..

[52] Bonetto G., Corradi M., Carraro S., Zanconato S., Alinovi R., Folesani G., Da Dalt L., Mutti A., Baraldi E. (2006). Longitudinal Monitoring of Lung Injury in Children after Acute Chlorine Exposure in a Swimming Pool. Am. J. Respir. Crit. Care Med..

[53] Kennedy M.D., Davidson W.J., Wong L.E., Traves S.L., Leigh R., Eves N.D. (2015). Airway inflammation, cough and athlete quality of life in elite female cross-country skiers: A longitudinal study. Scand. J. Med. Sci. Sports.

[54] Karjalainen E.M., Laitinen A., Sue-Chu M., Altraja A., Bjermer L., Laitinen L.A. (2000). Evidence of Airway Inflammation and Remodeling in Ski Athletes with and without Bronchial Hyperresponsiveness to Methacholine. Am. J. Respir. Crit. Care Med..

[55] Anderson S.D., Brannan J.D. (2003). Methods for “Indirect” Challenge Tests Including Exercise, Eucapnic Voluntary Hyperpnea, and Hypertonic Aerosols. Clin. Rev. Allergy Immunol..

[56] Perpiñá Tordera M., García Río F., Álvarez Gutierrez F.J., Cisneros Serrano C., Compte Torrero L., Entrenas Costa L.M., Melero Moreno C., Rodríguez Nieto M.J., Torrego Fernández A. (2013). Guidelines for the Study of Nonspecific Bronchial Hyperresponsiveness in Asthma. Arch. Bronconeumol..

[57] Sterk P.J., Bel E.H. (1989). Bronchial hyperresponsiveness: The need for a distinction between hypersensitivity and excessive airway narrowing. Eur. Respir. J..

[58] Thornton D.J., Sheehan J.K. (2004). From mucins to mucus: Toward a more coherent understanding of this essential barrier. Proc. Am. Thorac. Soc..

[59] Anderson S.D., Kippelen P. (2005). Exercise-induced bronchoconstriction: Pathogenesis. Curr. Allergy Asthma Rep..

[60] Heir T., Oseid S. (1994). Self-reported asthma and exercise-induced asthma symptoms in high-level competitive cross-country skiers. Scand. J. Med. Sci. Sports.

[61] Hallstrand T.S., Debley J.S., Farin F.M., Henderson Jr W.R. (2007). Role of MUC5AC in the pathogenesis of exercise-induced bronchoconstriction. J. Allergy Clin. Immunol..

[62] Bonini M., Gramiccioni C., Fioretti D., Ruckert B., Rinaldi M., Akdis C., Todaro A., Palange P., Carlsen K.H., Pelliccia A. (2015). Asthma, allergy and the Olympics: A 12-year survey in elite athletes. Curr. Opin. Allergy Clin. Immunol..

[63] Dutau G. (2017). Sport, asthme et allergie. Rev. Fr. Allergol..

[64] (2020). Allergies|Inserm—La Science Pour la Santé. https://www.inserm.fr/information-en-sante/dossiers-information/allergies.

[65] OMS Réseau International des Autorités de Sécurité Sanitaire des Aliments (INFOSAN). https://www.who.int/foodsafety/fs_management/No_03_allergy_June06_fr.pdf.

[66] Vermeer P.D., Harson R., Einwalter L.A., Moninger T., Zabner J. (2003). Interleukin-9 Induces Goblet Cell Hyperplasia during Repair of Human Airway Epithelia. Am. J. Respir. Cell Mol. Biol..

[67] Mäki-Heikkilä R., Karjalainen J., Parkkari J., Valtonen M., Lehtimäki L. (2020). Asthma in Competitive Cross-Country Skiers: A Systematic Review and Meta-analysis. Sports Med..

[68] Nwaru B.I., Ekström M., Hasvold P., Wiklund F., Telg G., Janson C. (2020). Overuse of short-acting β2-agonists in asthma is associated with increased risk of exacerbation and mortality: A nationwide cohort study of the global SABINA programme. Eur. Respir. J..

[69] Johnson M., Busse W.W., Holgate S.T. (2000). Mechanisms of action of b2-adrenoceptor agonists. Asthma & Rhinitis.

[70] Barnes P.J. (1999). Effect of β agonists on inflammatory cells. J. Allergy Clin. Immunol..

[71] Perttunen H., Moilanen E., Zhang X., Barnes P.J., Kankaanranta H. (2008). β2-Agonists Potentiate Corticosteroid-Induced Neutrophil Survival. COPD.

[72] Anderson R., Theron A.J., Steel H.C., Durandt C., Tintinger G.R., Feldman C. (2014). The Beta-2-Adrenoreceptor Agonists, Formoterol and Indacaterol, but Not Salbutamol, Effectively Suppress the Reactivity of Human Neutrophils In Vitro. Mediat. Inflamm..

[73] Johnson M. (2006). Molecular mechanisms of β2-adrenergic receptor function, response, and regulation. J. Allergy Clin. Immunol..

[74] Nelson J.A., Strauss L., Skowronski M., Ciufo R., Novak R., McFadden E.R. (1998). Effect of long-term salmeterol treatment on exercise-induced asthma. N. Engl. J. Med..

[75] Kalra S., Swystun V.A., Bhagat R., Cockcroft D.W. (1996). Inhaled Corticosteroids Do Not Prevent the Development of Tolerance to the Bronchoprotective Effect of Salmeterol. Chest.

[76] Cockcroft D.W., Swystun V.A., Bhagat R. (1995). Interaction of inhaled beta 2 agonist and inhaled corticosteroid on airway responsiveness to allergen and methacholine. Am. J. Respir. Crit. Care Med..

[77] Dickinson J., McConnell AWhyte G. (2011). Diagnosis of exercise-induced bronchoconstriction: Eucapnic voluntary hyperpnoea challenges identify previously undiagnosed elite athletes with exercise-induced bronchoconstriction. Br. J. Sports Med..

[78] Guarnieri M., Balmes J.R. (2014). Outdoor air pollution and asthma. Lancet.

